# Reduced Cell-Free Mitochondrial DNA Levels Were Induced by Antipsychotics Treatment in First-Episode Patients With Schizophrenia

**DOI:** 10.3389/fpsyt.2021.652314

**Published:** 2021-07-07

**Authors:** Houxian Ouyang, Minfang Huang, Yongming Xu, Qin Yao, Xiangping Wu, Dongsheng Zhou

**Affiliations:** ^1^Department of Psychiatry, Ningbo Kangning Hospital, Ningbo, China; ^2^Department of Laboratory Diagnostics, Ningbo Kangning Hospital, Ningbo, China; ^3^Department of Pharmacology, Ningbo Kangning Hospital, Ningbo, China; ^4^Ningbo Key Laboratory of Sleep Medicine, Ningbo, China

**Keywords:** first-episode schizophrenia, cell-free mitochondrial DNA, positive and negative syndrome scale, clinical progression, antipsychotics

## Abstract

Cell-free mitochondrial DNA (cf-mtDNA) is a damage-associated molecular pattern that boosts the release of cytokines and induces the immune response of the body; therefore, it is closely related to mental diseases. This study aims to evaluate a potential link between cf-mtDNA and clinical progression in first-episode patients with schizophrenia. In this study, plasma cf-mtDNA levels in 34 first-episode patients with schizophrenia before and after 8 weeks of antipsychotic treatment were examined. In addition, the clinical progression of first-episode schizophrenia was assessed using the Positive and Negative Syndrome Scale (PANSS). The copy number changes in the plasma cf-mtDNA (Δcf-mtDNA) were significantly correlated with changes in the PANSS scale scores (ΔPANSS) in first-episode patients with schizophrenia (ΔPANSS total score, *P* = 0.002; ΔPANSS positive score, *P* = 0.01). Plasma cf-mtDNA may represent a relevant tool in the future to assist in the assessment of clinical progression in first-episode patients with schizophrenia.

## Introduction

Schizophrenia causes great suffering in afflicted patients, induces a severe burden to affected families, yields a high medical expenditure, and has gradually become a significant social problem (1). The diagnosis and treatment of schizophrenia are primarily dependent on the clinical manifestations. The most critical period for effective control and treatment of schizophrenia is the first episode.

Excess oxidative stress and immune status disequilibrium could be involved in the pathophysiology and represent an underlying mechanism that explains different clinical symptoms of schizophrenia (2–4). Although some cytokines and reactive oxygen species have already been described for schizophrenia (5–8), the clinical utility of those findings remains limited, and few of them have been applied to clinical practice. Excess oxidative stress causes mitochondrial damage and leads to the release of mtDNA (9–11). MtDNA is more stable and more applicable compared with some oxidative stress laboratory parameters (e.g., hydrogen peroxide, hydroxyl radicals, and others) (12). From a biological point of view, this is interesting. MtDNA containing unmethylated inflammatory CpG motifs is released into the circulatory system and activates the toll-like receptor 9 (TLR-9) signaling pathways and NLRP3 (NOD-, LRR-, and pyrin domain-containing 3) inflammasome, which in turn boosts the release of cytokines (e.g., interleukin 6, interleukin 18, Interleukin 1β, and others). This induces the immune response of the body (3, 13–16).

In summary, cell-free mitochondrial DNA (cf-mtDNA) reflects both the degree of the oxidative stress condition and immune homeostasis. Furthermore, compared with intracellular mtDNA, cf-mtDNA is easier to obtain, which eliminates complex cell selection operations and facilitates the stability of detection.

This study aims to investigate the plasma cf-mtDNA levels in drug-free first-episode patients with schizophrenia. The putative changes in the plasma cf-mtDNA levels during first-episode patients with schizophrenia are also studied using antipsychotic treatments.

## Materials and Methods

### Participants

This study was approved by the Ethics Committee of the Ningbo Kangning Hospital. In the case group, inpatients (*N* = 34) who were diagnosed with first-episode schizophrenia as assessed by the Diagnostic and Statistical Manual of Mental Disorders, 5th Edition (DSM-5) were recruited from Ningbo Kangning Hospital. The subjects had not taken antipsychotics, antidepressants, antimanic, or antiepileptics for at least 2 weeks prior to enrollment. The initial clinical dose of aripiprazole was 10 mg/d, and the dose was gradually increased to a steady concentration of 20 mg/d in the first-episode patients with schizophrenia. Healthy controls (HCs) (*N* = 28) were matched for age, sex, and body mass index (BMI) to the first-episode patients with schizophrenia. The healthy control participants also met the following criteria: (a) no history of neuropsychiatric disorders; (b) did not meet any of the DSM-5 criteria for the diagnosis of mental disease; (c) no prescription medications (antipsychotics, antidepressants, antimanic, or antiepileptics) for at least 2 weeks. Furthermore, any subjects with physical conditions, including severe or secondary somatic disease (e.g., acute infection, autoimmune diseases, tumors, and mitochondrial diseases) or a history of smoking, alcohol consumption, or drug abuse, were excluded from this study. Female patients who were pregnant or lactating were also excluded. For all of the subjects, a questionnaire survey was conducted by trained research staff to collect general information, demographic characteristics, and medical conditions. Subjects were excluded if confounding factors [e.g., death of husband/wife, divorce, death of a close family member (not spouse), major personal illness, major illness of a family member, sexual difficulties, marriage, changes in financial condition, and other factors] were found in the survey data using the Social Readjustment Rating Scale (score > 150).

### Primer Design

The Mitochondrial NADH dehydrogenase subunit 1 (*MT-ND1*) gene was used as the target gene for cf-mtDNA copy number detection, and the hemoglobin (*HGB*) gene was used as the reference gene. Specific primer sequences were designed using Primer Premier 5.0 software. The *MT-ND1* gene was amplified and quantified using the following primers: 5′-CCCTAAAACCCGCCACATCT-3′ (forward) and 5′-GAGCGATGGTGAGAGCTAAGGT-3′ (reverse). The *HGB* gene was amplified and quantified using the following primers: 5′-GCTTCTGACACAACTGTGTTCACTAGC-3′ (forward) and 5′-CACCAACTTCATCCACGTTCACC-3′ (reverse).

### Quantification of Plasma cf-mtDNA

Venous blood was collected in a tube containing EDTA-K_2_ anticoagulant in the morning and centrifuged at 2,000 g for 10 min. The supernatant was centrifuged (16,000 g × 10 min at 4°C, Anhui Zhongke Zhongjia Instrument Co., Ltd., China) and stored at −80°C. The circulating cell-free DNA in the plasma was extracted using a Mag-Bind®cfDNA Kit (Omega Bio-tek Inc., USA). The PCR thermocycler (Bio-Rad, USA) parameters included the following: 95°C for 3 min, 35 cycles of 95°C for 30 s, 57°C for 30 s, and 72°C for 30 s; and a final extension at 72°C for 8 min. Each 25 μl of PCR reaction contained 2.5 μl of 10 × PCR Buffer (Sangon Biotech Co., Ltd., China), 18.3 μl of PCR-grade H_2_O, 0.5 μl of each primer (Sangon Biotech Co., Ltd., China), and 0.5 μl of the sample DNA.

The centrifuged supernatant of the blood samples was used as a template for quantitative real-time PCR (Roche Diagnostics, Germany). The real-time PCR parameters included the following: 95°C for 3 min; and 45 cycles of 95°C for 15 s, 57°C for 20 s, and 72°C for 30 s. Each 10 μl real-time PCR reaction contained 2 × SybrGreen qPCR Master Mix (Sangon Biotech Co., Ltd., China), 3.6 μl of PCR-grade H_2_O, 0.2 μl of each primer (10 μM) (Sangon Biotech Co., Ltd., China), and 1.0 μl of the sample DNA (centrifuged supernatant of blood samples diluted five times). cf-mtDNA copy numbers were normalized to that of the nDNA control gene, *HGB*, using PCR products for the standard curve using pMD 18-T Vector transformation technology (TAKARA Bio Inc., Japan).

### Positive and Negative Syndrome Scale (PANSS) Assessment

The PANSS assessment was conducted in all of the subjects at both enrollments and the end of the eighth week of the study. Here, the PANSS total score (PANSS-T) was composed of the PANSS positive score (PANSS-P), the PANSS negative score (PANSS-N), and the PANSS general psychopathology score (PANSS-G). The PANSS scale was evaluated using two experienced clinicians. The reliability was determined using the internal consistency test (kappa > 0.90).

### Statistical Analysis

All of the statistical analyses were performed using the SPSS 19.0 (SPSS Inc., USA). The normal distributions of the year, the BMI, the cf-mtDNA copy number, and the PANSS scores were assessed using the Kolmogorov–Smirnov test. A paired *t*-test was used to assess before and after drug treatment, and the group *t*-test was used to evaluate the comparison between the case group and the healthy control group. The Wilcoxon non-parametric test was used for non-normal distribution analyses. The chi-square test was used to assess the gender distribution between the two groups. The influence of gender, age, and BMI on the plasma cf-mtDNA levels was tested using linear regression. A correlation analysis was used to evaluate the relationship between the plasma cf-mtDNA copy number and the PANSS scores. *P* < 0.05 was considered to be statistically significant.

## Results

The demographic characteristics of all of the subjects are shown in Table 1. No significant differences were observed in gender, age, and BMI between the first-episode patients with schizophrenia and the HCs (χ^2^ = 0.01, *P* = 0.97; *t* = 0.83, *P* = 0.41; *t* = 0.39, *P* = 0.70). The linear regression analysis on all of the samples showed that gender, age, and BMI had no significant effect on the plasma cf-mtDNA levels (*F* = 0.30, *P* = 0.83).

**Table 1:** Demographic characteristics of first-episode patients with schizophrenia and healthy controls (HCs) enrolled in the study.

**Index**	**First-episode schizophrenic patients (*N* = 34)**	**Healthy controls (*N* = 28)**	**Statistical value**
Age (year)	34.8 ± 8.3	36.7 ± 9.7	*t* = 0.83, *P* = 0.41
Gender (Males/Females)	(12/22)	(10/18)	*χ^2^* = 0.01, *P* = 0.97
BMI (Kg/m^2^)	21.8 ± 1.8	22.0 ± 1.9	*t* = 0.39, *P* = 0.70

*A linear regression analysis on all the subjects was used to evaluate the effects of demographic characteristics on plasma cell-free mitochondrial DNA (cf-mtDNA) levels with gender, age, and body mass index (BMI) included as covariates (F = 0.30, P = 0.83)*.

**Figure 1:**
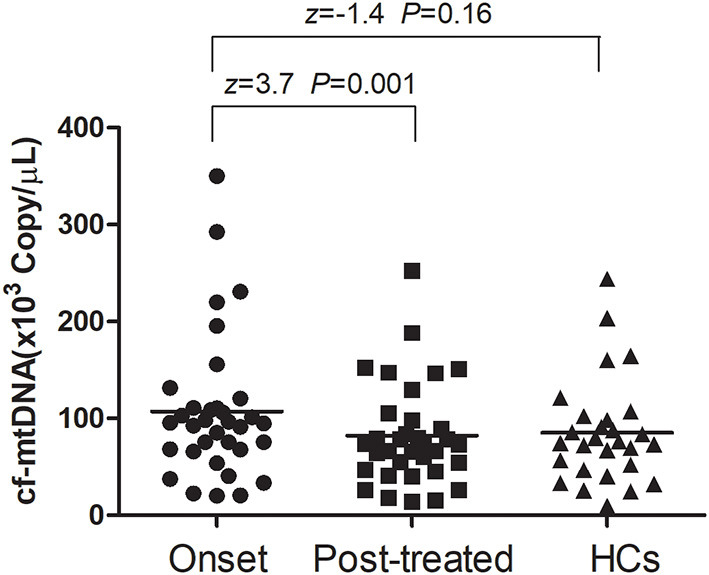
Comparison of the plasma cell-free mitochondrial DNA (cf-mtDNA) levels among the different groups. The Wilcoxon Mann–Whitney test was used for the data analysis between the onset and healthy controls (HCs) groups (*z* = −1.4, *P* = 0.16). The Wilcoxon signed-rank test was used for the data analysis between the onset and posttreated groups (*z* = 3.7, *P* = 0.001).

**Figure 2:**
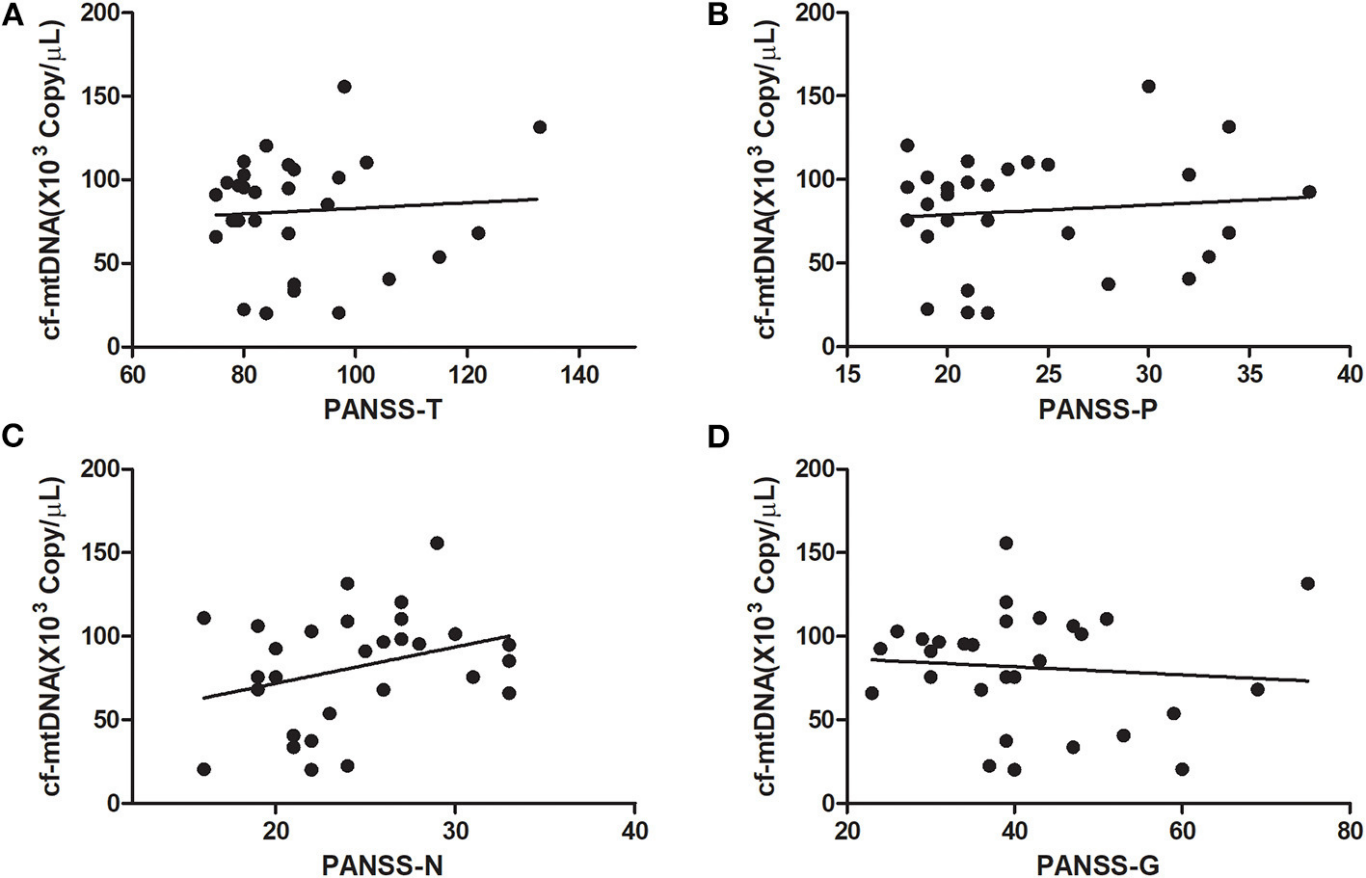
Correlations between the cf-mtDNA copy number and components of the PANSS scores in first-episode patients with schizophrenia (in baseline). **(A)** *r* = 0.04, *P* = 0.83; **(B)** *r* = 0.23, *P* = 0.19; **(C)** *r* = 0.05, *P* = 0.77; **(D)** *r* = −0.04, *P* = 0.84.

**Figure 3:**
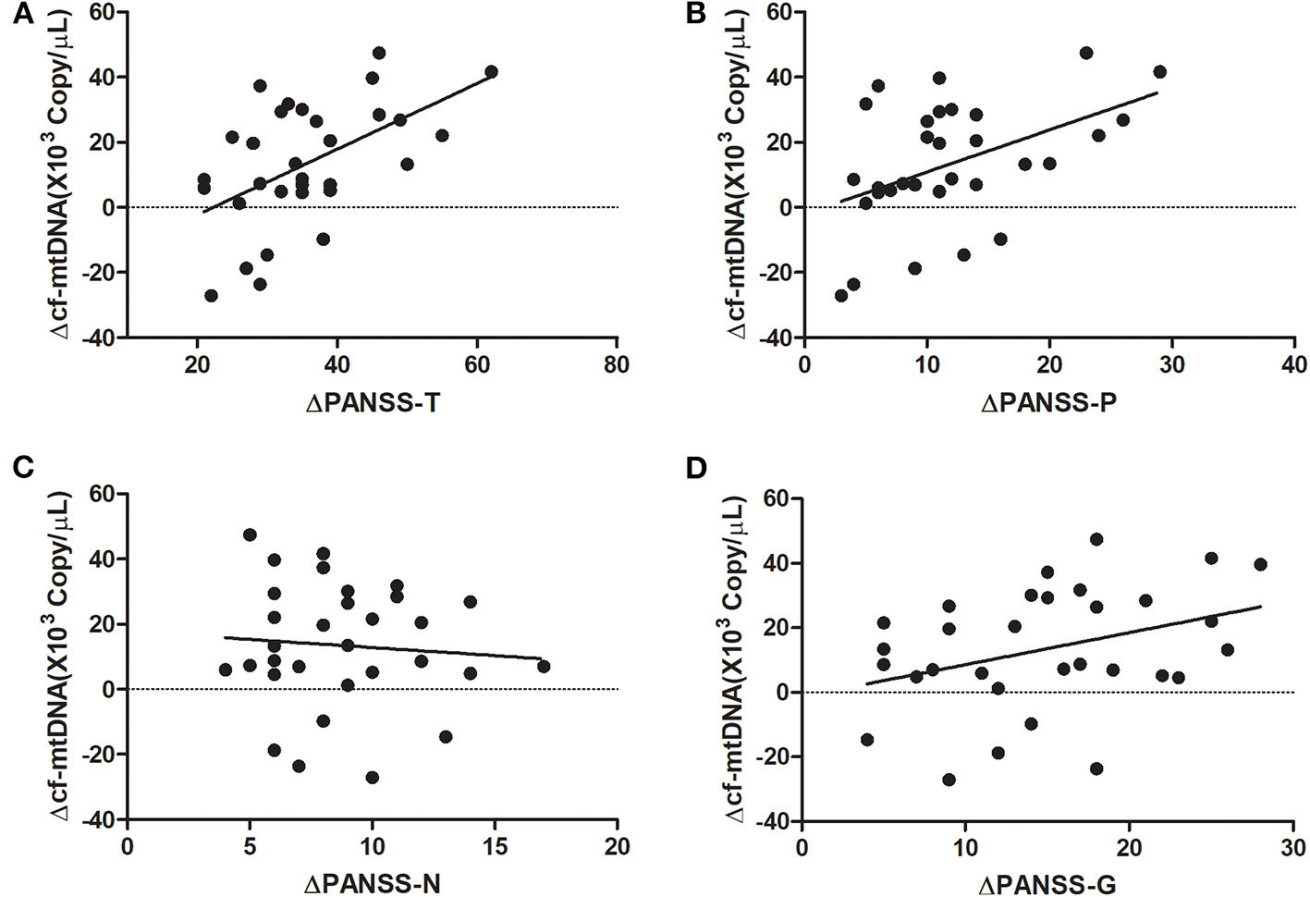
Correlation analysis of the copy number changes in the plasma cf-mtDNA (Δcf-mtDNA) with components of the PANSS scores in first-episode patients with schizophrenia (after 8 weeks of antipsychotic treatment). **(A)** *r* = 0.53, *P* = 0.002; **(B)** *r* = 0.46, *P* = 0.01; **(C)** *r* = −0.08, *P* = 0.66; **(D)** *r* = 0.35, *P* = 0.055.

At baseline, the plasma cf-mtDNA copy number in the first-episode patients with schizophrenia showed no significant difference compared to the HCs (*z* = −1.4, *P* = 0.16), and they were reduced by the use of the antipsychotic treatments (*z* = 3.7, *P* = 0.001; Table 2 and Figure 1). After 8 weeks of antipsychotic treatments, the cf-mtDNA, PANSS-T, PANSS-P, PANSS-N, and PANSS-G were each significantly different before and after treatment (*z* = 3.7, *P* = 0.001; *z* = 5.1, *P* < 0.001; *z* = 5.1, *P* < 0.001; *t* = 16.1, *P* < 0.001; *z* = 5.1, *P* < 0.001) (Table 2). The correlation analysis showed that there was no significant correlation between the plasma cf-mtDNA copy number and the clinical progression (PANSS scores) in first-episode patients with schizophrenia (PANSS-T: *r* = 0.04, *P* = 0.83; PANSS-P: *r* = 0.23, *P* = 0.19; PANSS-N: *r* = 0.05, *P* = 0.77; PANSS-G: *r* = −0.04, *P* = 0.84) (Figure 2). Interestingly, significant correlations were observed between copy number changes in the plasma cf-mtDNA (Δcf-mtDNA) and partial symptom improvement (ΔPANSS-T: *r* = 0.53, *P* = 0.002; ΔPANSS-P: *r* = 0.46, *P* = 0.01) (Figure 3).

**Table 2:** Plasma cf-mtDNA detection and the Positive and Negative Syndrome Scale (PANSS) assessment of the subjects.

**Index**	**First-episode schizophrenic patients**		**Healthy controls**
	**Onset**	**Post-treated**	
mtDNA (×10^3^ Copy/μL)*	95.1 (67.3, 113.3)	73.8 (46.7, 100.0)	75.4 (48.1, 101.2)
PANSS-T*	87 (80, 97)	54 ± 10.4	
PANSS-P*	22.5 (20, 30)	12.4 ± 4.3	
PANSS-N*	23.8 ± 5.1	15.2 ± 5.1	
PANSS-G*	41.4 ± 12.6	27.5 (21.75, 30.25)	

*Significant P-values are highlighted via asterisks. PANSS-T, PANSS total score; PANSS-P, PANSS positive score; PANSS-N, PANSS negative score; PANSS-G, PANSS general psychopathology score. Normally distributed data are expressed as means ± SDs, while non-normally distributed data are expressed as the interquartile range*.

## Discussion

In the present study, consistent plasma cf-mtDNA levels were observed between the first-episode patients with schizophrenia and the HCs. After antipsychotic treatment, the plasma cf-mtDNA levels were reduced and related to the PANSS scores.

It was found that first-episode patients with schizophrenia showed similar plasma cf-mtDNA levels to those of the HCs. This result suggested that this parameter had no association with schizophrenic risk. This result was similar to that of the previous study (17). The results of this study indicated that the plasma cf-mtDNA may not be useful as a potential diagnostic biomarker in this context. Even so, the utility of cf-mtDNA based on mtDNA being known to exhibit damage-associated molecular patterns (DAMPs) was further investigated. Such detailed data regarding changes in plasma cf-mtDNA levels have been lacking for schizophrenic clinical progression. Therefore, whether the plasma cf-mtDNA levels were indicative of clinical progression in such patients was investigated. Clinical antipsychotic medications were given to first-episode patients with schizophrenia, and plasma cf-mtDNA levels were found to be significantly decreased compared with those in pretreated patients. Coincidentally, these results were similar to a recent study that reported that the plasma cf-mtDNA levels were significantly lower in depressed adult patients compared to HCs (17–19). In contrast, He et al. (20) reported that there were no changes in the mtDNA copy number of peripheral blood leukocytes in major depressive disorder. The probable reasons for the discrepancy are the disturbing effect of somatic diseases (e.g., mitochondrial disease), different sample handling, or the effect of medications. Combined with changes in the PANSS scores, this study revealed that changes in the plasma cf-mtDNA levels were associated with clinical progression, as changes in the plasma copy numbers were positively correlated with both the PANSS-P and PANSS-T. TLR-9-induced upregulation of nuclear factor kappa B (NFκB) *via* the primary response pathway for myelination differentiation leads to the release of cytokines, such as interleukin 6 (IL-6) (14, 15). IL-6 is closely related to PANSS-P scores in patients with schizophrenia (21, 22). The findings of this study corroborate this correlation and also illustrate the value of cf-mtDNA as a potential link of the PANSS scores in first-episode patients with schizophrenia.

The present study had some limitations. First, the sample size was very small, and the average age of the patients was older than expected. Second, there was a lack of other measurements in addition to the cf-mtDNA (e.g., related putative biomarkers, DAMPs, and cytokines). Third, the study (exclusion of smoking, alcohol consumption, or drug abuse) may not be generalized to all patients with schizophrenia. Fourth, the reasons for the cf-mtDNA levels changes remain unclear and require further research.

In summary, the results suggested that plasma cf-mtDNA levels were reduced in drug-free first-episode patients with schizophrenia after 8 weeks of antipsychotic treatment, and the change in the PANSS scores was associated with the change in the plasma cf-mtDNA levels. In the future, the use of plasma cf-mtDNA levels may be a relevant tool to assist in the assessment of clinical progression in first-episode patients with schizophrenia.

## Data Availability Statement

The original contributions presented in the study are included in the article, further inquiries can be directed to the corresponding author.

## Ethics Statement

The studies involving human participants were reviewed and approved by Ethics Committee of Ningbo Kangning Hospital. The patients/participants provided their written informed consent to participate in this study.

## Author Contributions

DZ and HO: conceptualization and validation. HO: methodology and writing the original draft preparation. MH and QY: software. YX: formal analysis. DZ, XW, and MH: resources. DZ: writing the review and editing, and project administration. All authors have read and agreed to the published version of the manuscript.

## Conflict of Interest

The authors declare that the research was conducted in the absence of any commercial or financial relationships that could be construed as a potential conflict of interest.
